# Congenital Talipes Equinovarus Management and Outcomes: The Experiences of Pediatric Tertiary Centers in Abha, Saudi Arabia

**DOI:** 10.7759/cureus.43264

**Published:** 2023-08-10

**Authors:** Saleh M Kardm, Ahmed S Al Zomia, Ali A Alqahtani, Faisal M Al Fae, Ibrahim A Al Zehefa, Yazeed S Alshahrani, Fahad A A AlShehri, Abdulrhman M Alqarni, Abdulrahman S Alqahtani

**Affiliations:** 1 Department of Orthopedics, Faculty of Medicine, Najran University, Najran, SAU; 2 College of Medicine, King Khalid University, Abha, SAU; 3 College of Medicine, Umm Al-Qura University, Makkah, SAU

**Keywords:** pediatric, saudi arabia, ponseti, consanguineous, outcome, management, risk factors, prevalence, congenital talipes equinovarus, clubfoot

## Abstract

Background: Congenital talipes equinovarus (CTEV), also known as clubfoot, describes a range of foot abnormalities usually present at birth (congenital) in which a baby’s foot is twisted out of shape or position. In clubfoot, tendons are shorter than usual. Clubfoot is a fairly common birth defect and is usually an isolated problem for an otherwise healthy newborn.

Aim: This study aimed to investigate the experiences of pediatric tertiary centers in Abha, Saudi Arabia, regarding the management, frequency, treatment options, and outcomes of CTEV.

Methods: A retrospective chart review of pediatric patients with clubfoot was conducted to evaluate the number of cases, treatment options, and outcomes at Abha Maternity and Children Hospital and Khamis Mushait Maternity and Children Hospital. Data were extracted independently using prestructured data extraction forms. The collected data included demographic and medical information, family history, clinico-epidemiological information, risk factors, management options, and complications of clubfoot.

Results: The study included 89 children with CTEV from the target hospitals. Their ages ranged from 20 days to six years, with a mean age of 10.5 ± 14.5 months. Of these, 57 (64%) were male. CTEV was unilateral in 53 (59.5%) cases and bilateral in 36 (40.5%) cases. The majority of the cases had isolated CTEV. Nearly all cases had Ponseti casting with a tendo-Achilles tenotomy (TAT) surgical procedure. Patient follow-up ranged from one week to three years, with an average follow-up of 3.1 months. Only three (3.4%) cases experienced recurrence of deformity after management.

Conclusion: Ponseti casting with the tendo-Achilles tenotomy approach emerged as the most commonly employed management option, demonstrating a low rate of recurrence.

## Introduction

Congenital talipes equinovarus (CTEV), also called clubfoot, is a congenital disability featuring cavus, adductus, varus, and equinus leg abnormalities [1]. The deformity may be unilateral or bilateral, and it is one of the most common congenital musculoskeletal disabilities [2]. This is different from other positional foot anomalies because the foot is rigid and does not correct with passive movement [3]. Clubfoot is the second most dominant congenital anomaly among neonates, after hip dysplasia. Out of every 1,000 infants, one to two are born with this malformation [4]. The incidence of CTEV is nearly doubled among boys, compared to girls, and is mainly bilateral [4,5]. Although infant clubfoot ranges in severity, deviations from healthy feet are clearly obvious to laypeople. The malformed foot typically points inward, with a deep crease on the bottom [6].

The Achilles tendon is shortened, and the heel is soft and turned up mainly in cases of unilateral clubfoot. The calf on the affected foot becomes thinner than that on the unaffected foot. Clubfoot is an abnormality that a professional can identify by its distinctive characteristics, in which the foot shifts inward and downward in relation to the talus bone [7-9]. Its typical characteristics include equinus foot, shortened Achilles tendon, downward sloping foot (pes adductus), hollow foot with elevated longitudinal arch (pes excavatus), and varus position of the calcaneus (“O position”) [10,11].

There is no specific cause for CTEV [12]. Several explanations have been suggested, including those related to the circulatory system, viruses, genetics, anatomy, compartment syndrome, environmental influences, and the position of the fetus in the womb [13]. The existence of a neuromuscular foundation for this illness is still disputable. Some studies have revealed anomalies in the ultrastructure and intracellular organization of clubfoot muscle samples, while others have not [12,13]. Studies on children with clubfoot support a single significant genetic component, and twin observations can help identify whether this is the primary genetic cause. Monozygotic twins have higher rates of having CTEV than dizygotic twins. A recent study from the Danish Twin Registry discovered that there is a one in three chance that a second monozygotic twin will also have clubfoot, indicating that causes other than genetics may be involved [12,14,15]. In a systematic review, evidence from families and twins suggests a genetic component in clubfoot, although the specific inheritance patterns remain unclear [15].

Idiopathic CTEV is currently treated with serial casting using the Ponseti method to gradually rectify the deformity, followed by years of bracing to maintain the repair [16]. Although the Ponseti approach has reportedly been effective in treating idiopathic CTEV, to our knowledge, there have been no reports of its use in non-idiopathic CTEV. On the other hand, patients with non-idiopathic CTEV are sometimes primarily treated with extensive surgical releases, since it is believed that the abnormalities in these patients are too rigid to be corrected with casting alone [16]. Therefore, the second purpose of this study is to determine whether non-idiopathic CTEV (caused by a specific known underlying cause or associated with other medical conditions) could be corrected using the Ponseti method with a high success rate and low recurrence risk, similar to idiopathic CTEV [3].

## Materials and methods

This study involved a retrospective chart review to assess the prevalence of clubfoot cases, treatment options, and patient outcomes in pediatric patients at Abha Maternity and Children Hospital and Khamis Mushait Maternity and Children Hospital. Patient data were obtained from medical records spanning from August 2022 to March 2023. To ensure accuracy, data extraction was carried out independently using prestructured data extraction forms. The collected information encompassed various aspects, including demographics, medical history, family history of clubfoot, clinico-epidemiological data, risk factors, treatment options, and any complications observed. Cases with incomplete clinical data and instances where communication with parents was unsuccessful were excluded from the analysis to maintain data integrity.

Data analysis

We employed numbers and frequencies to describe study variables such as personal data, family history, clinico-epidemiological pattern of CTEV, factors associated with CTEV, management options, and clinical outcomes. For numerical data that exhibited a normal distribution, we used the mean and standard deviation (SD). To investigate the factors associated with clinical outcomes in patients with CTEV, we conducted cross-tabulation and subsequently analyzed the results using Pearson’s chi-square test for significance. In case of violation of the chi-square assumption, the Monte Carlo test was used instead. In cases where the assumptions of the chi-square test were violated, we utilized the exact probability test as an alternative. All statistical analyses were performed using the Statistical Package for the Social Sciences (SPSS) version 21 (IBM SPSS Statistics, Armonk, NY, USA), ensuring accurate and reliable data analysis. The statistical methods used in the study were two-tailed, and a significance level (alpha coefficient) of 0.05 was adopted.

Ethics

This research was conducted in accordance with the ethical principles and guidelines outlined in the Helsinki Declaration. Approval for the study protocol was obtained from the Ministry of Health, Aseer Regional Committee for Research Ethics (approval number: REC-13-10-2022). All participants provided informed consent before their inclusion in the study.

## Results

A total of 89 children with CTEV were included in the study. Their ages ranged from 20 days to six years, with a mean age of 10.5 ± 14.5 months. Of these, 57 (64%) were males, and only three had a family history of CTEV (Table 1).

**Table 1:** Personal data of cases with CTEV at maternity and children’s hospitals in Abha, Saudi Arabia CTEV: congenital talipes equinovarus

Personal data	Number	%
Age of patient		
<6 months	47	52.8%
6 months to 1 year	26	29.2%
>1 year	16	18%
Gender		
Male	57	64%
Female	32	36%
Family history of clubfoot		
Yes	3	3.4%
No	86	96.6%

Table 2 shows the clinical features of CTEV and its associated conditions among the studied cases. The abnormality was unilateral in 53 (59.5%) cases and bilateral in 36 (40.5%) cases. A vast majority of the cases had isolated CTEV, while 12 (13.5%) of the cases were associated with other medical conditions, including Achilles tendon tightness (one case), congenital hydrocephalus with hydronephrosis and paraplegia (one case), Down syndrome (one case), dwarfism (one case), dysmorphic features with a rash (one case), right hip dysplasia (one case), and ventricular septal defect (VSD) (one case).

**Table 2:** CTEV clinical features and associated conditions among study cases CTEV: congenital talipes equinovarus, VSD: ventricular septal defect

CTEV data	Number	%
Side of CTEV		
Right	34	38.2%
Left	19	21.3%
Bilateral	36	40.5%
CTEV association		
Isolated	87	97.8%
Not isolated	2	2.2%
Associated with other medical conditions		
No	77	86.5%
Yes	5	5.6%
Achilles tendon tightness	1	1.1%
Congenital hydrocephalus with hydronephrosis and paraplegia	1	1.1%
Down syndrome	1	1.1%
Dwarfism	1	1.1%
Dysmorphic features with rash	1	1.1%
Right hip dysplasia	1	1.1%
VSD	1	1.1%

Table 3 shows the risk factors for CTEV among the studied cases at maternity and children’s hospitals in Abha, Saudi Arabia. Parental consanguinity was confirmed among 34 (38.2%) cases. Only two cases were suspected as preterm, 83 (93.3%) cases involved normal vaginal delivery (NVD), and most gestations (97.8%) were singleton births.

**Table 3:** Risk factors of CTEV among study cases at maternity and children’s hospitals in Abha, Saudi Arabia CTEV: congenital talipes equinovarus, NVD: normal vaginal delivery, CS: cesarean section

Factors	Number	%
Parents’ consanguinity		
Yes	34	38.2%
No	55	61.8%
Gestational age		
Full term	87	97.8%
Unknown	2	2.2%
Mode of delivery		
NVD	83	93.3%
CS	6	6.7%
Number of gestations		
One	87	97.8%
More than one	2	2.2%

Table 4 shows the management options and clinical outcomes of CTEV cases at maternity and children’s hospitals in Abha, Saudi Arabia. Nearly all patients (88, 98.9%) received Ponseti casting with tendo-Achilles tenotomy (TAT) surgical procedures. Achilles lengthening was performed for nearly all cases (88, 98.9%). Patients’ follow-up periods ranged from one year to three years, with an average of 3.1 months. Only three (3.4%) cases experienced a recurrence of the deformity after management.

**Table 4:** Management options and clinical outcomes of CTEV cases in maternity and children’s hospitals in Abha, Saudi Arabia CTEV: congenital talipes equinovarus, TAT: tendo-Achilles tenotomy, SD: standard deviation

Management and outcomes	Number	%
Ponseti casting		
Yes	88	98.9%
No	1	1.1%
Achilles lengthening		
Yes	88	98.9%
No	1	1.1%
Surgical procedure		
TAT	89	100%
Complications		
None	86	96.6%
Recurrent deformity	3	3.4%
Follow-up period (months)		
Range	1 week to 3 years	
Mean ± SD	3.1 ± 4.0	

Table 5 illustrates the factors associated with the clinical outcomes of CTEV cases in maternity and children’s hospitals in Abha, Saudi Arabia. Among all factors, only mode of delivery showed significant association with complications, as deformity recurrence was reported in 16.7% of cases who were born by cesarean section (CS), while it was reported in 2.4% of cases who were born by NVD (P = 0.049).

**Table 5:** Factors associated with clinical outcomes of CTEV in maternity and children’s hospitals in Abha, Saudi Arabia *p < 0.05 is considered significant (exact probability test). CTEV: congenital talipes equinovarus, NVD: normal vaginal delivery, CS: cesarean section

Factors	Complications				p-value
	None		Recurrent deformity		
	Number	%	Number	%	
Age of patients					0.723
<6 months	46	97.9%	1	2.1%	
6 months to 1 year	25	96.2%	1	3.8%	
>1 year	15	93.8%	1	6.3%	
Gender					0.923
Male	55	96.5%	2	3.5%	
Female	31	96.9%	1	3.1%	
Association with other medical conditions					0.487
Yes	12	100%	0	0%	
No	74	96.1%	3	3.9%	
Gestational age					0.789
Full term	84	96.6%	3	3.4%	
Unknown	2	100%	0	0%	
Mode of delivery					0.049*
NVD	81	97.6%	2	2.4%	
CS	5	83.3%	1	16.7%	
Number of gestations					0.789
1 baby	84	96.6%	3	3.4%	
>1 baby	2	100%	0	0%	
Side of CTEV					0.081
Right	31	91.2%	3	8.8%	
Left	19	100%	0	0%	
Bilateral	36	100%	0	0%	
Ponseti casting					0.851
Yes	85	96.6%	3	3.4%	
No	1	100%	0	0%	
Achilles lengthening					0.851
Yes	85	96.6%	3	3.4%	
No	1	100%	0	0%	

## Discussion

This study reveals that nearly two-thirds of the cases were males, and only three cases had a family history of CTEV. Regarding clinical features and associated conditions, the deformity was unilateral in more than half of the cases, and most of the CTEV cases were isolated. A few of the cases were associated with other medical conditions, such as Achilles tendon tightness, congenital hydrocephalus with hydronephrosis and paraplegia, Down syndrome, dwarfism, dysmorphic features with a rash, right hip dysplasia, and VSD. Commonly known as clubfoot, CTEV is a complex deformity of the foot and ankle present at birth [7]. The condition affects the structure and function of the feet, often resulting in walking difficulties and other complications [3,5]. Treatment for CTEV typically begins soon after birth, with a focus on conservative methods, such as the Ponseti technique. Surgical intervention may be necessary in some cases to achieve proper foot alignment and improve the overall quality of life for the affected individual [5,15].

In the literature, most cases of CTEV were isolated and idiopathic, whereas about one-fifth of the cases were associated with syndromic conditions (distal arthrogryposis, congenital myotonic dystrophy, myelomeningocele, amniotic band sequence, or other genetic syndromes, such as trisomy 18 or chromosome 22q11 deletion syndrome) [16]. In addition, a male-to-female ratio of 2:1 has been reported in isolated cases [17,18]. McConnell et al. [19] found that males were twice as likely to have clubfoot, and half of the cases had bilateral clubfoot. There was no significant difference in the rate of left versus right clubfoot. Infant and maternal characteristics that were significantly associated with clubfoot included breech presentation and old maternal age at conception. Other studies revealed other risk factors for clubfoot, including male gender [20-22], maternal smoking [23-26], maternal age [24], maternal marital status [22], maternal education [23,25], and maternal diabetes [25,26].

In this study, we found that parental consanguinity existed in more than one-third of the cases, two preterm births were reported, an NVD was reported for most cases, and almost all cases were singleton births.

Regarding the management options and clinical outcomes of CTEV, nearly all cases had Ponseti casting with a TAT surgical procedure. Achilles lengthening was also performed for nearly all cases. Only three cases experienced a recurrence of deformity after management. Haft et al. [27] reported that 41% of their cases experienced recurrence, which is much higher than the rate found in the current study. In addition, van Praag et al. [28] reported that up to 40% of patients with idiopathic clubfoot who were treated with the Ponseti method experienced a recurrence of deformity. Halanski et al. [29] reported that after Ponseti treatment, 19%-40% of patients needed further treatment for recurrence.

Strengths and limitations

This study presents valuable findings on the management and outcomes of CTEV in pediatric patients. However, several limitations should be acknowledged. The retrospective design may introduce biases and limitations due to data collection from past medical records, potentially leading to missing or incomplete information. The sample size may be limited, impacting the study’s statistical power and generalizability. Additionally, the study focused on specific hospitals within the same region, which may not fully represent the broader population. There might be unmeasured confounding factors, and causal relationships are challenging to establish in an observational study. While this study provides valuable insights, future research should consider prospective designs, larger sample sizes, and multicenter collaborations to address these limitations and strengthen the validity and applicability of the results.

## Conclusions

In conclusion, the current study showed that CTEV was frequent among babies of different age groups. A high prevalence was detected among male babies and in cases with parental consanguinity, but there was no association with the mode of delivery or the number of gestations. Most cases had a unilateral deformity, which was isolated and associated with some medical conditions. Ponseti casting using the TAT approach was the most frequently used management option with low recurrence rates.
